# The sucrose transporter MdSUT4.1 participates in the regulation of fruit sugar accumulation in apple

**DOI:** 10.1186/s12870-020-02406-3

**Published:** 2020-05-06

**Authors:** Qian Peng, Yaming Cai, Enhui Lai, Masayoshi Nakamura, Liao Liao, Beibei Zheng, Collins Ogutu, Sylvia Cherono, Yuepeng Han

**Affiliations:** 1 CAS Key Laboratory of Plant Germplasm Enhancement and Specialty Agriculture, Wuhan Botanical Garden, The Innovative Academy of Seed Design, Chinese Academy of Sciences, Wuhan, 430074 China; 2 University of Chinese Academy of Sciences, 19A Yuquanlu, Beijing, 100049 China; 3 Institute of Transformative Bio-Molecules, Nagoya University, Nagoya, Japan; 4 Center of Economic Botany, Core Botanical Gardens, Chinese Academy of Sciences, Wuhan, 430074 China; 5 Sino-African Joint Research Center, Chinese Academy of Sciences, Wuhan, 430074 China

**Keywords:** *Malus x domestica*, Gene-tagged marker, *SUT*, Tonoplast localization, Fruit sweetness

## Abstract

**Background:**

Sugar content is an important determinant of fruit sweetness, but details on the complex molecular mechanism underlying fruit sugar accumulation remain scarce. Here, we report the role of sucrose transporter (SUT) family in regulating fruit sugar accumulation in apple.

**Results:**

Gene-tagged markers were developed to conduct candidate gene-based association study, and an *SUT4* member *MdSUT4.1* was found to be significantly associated with fruit sugar accumulation. *MdSUT4.1* encodes a tonoplast localized protein and its expression level had a negative correlation with fruit sugar content. Overexpression of *MdSUT4.1* in strawberry and apple callus had an overall negative impact on sugar accumulation, suggesting that it functions to remobilize sugar out of the vacuole. In addition, *MdSUT4.1* is located on chromosomal region harboring a previously reported QTL for sugar content, suggesting that it is a candidate gene for fruit sugar accumulation in apple.

**Conclusions:**

*MdSUT4.1* is involved in the regulation of fruit sugar accumulation in apple. This study is not only helpful for understanding the complex mechanism of fruit sugar accumulation, but it also provides molecular tools for genetic improvement of fruit quality in breeding programs of apple.

## Background

Sucrose is a major product of photosynthesis in plants, and the most common form of carbohydrate transported through the phloem from source leaves to sink organs such as roots and fruits. Extensive research has been conducted in the last three decades on sucrose transport in plants and sucrose transporters (SUTs) are deemed to be essential for the export and efficient movement of sucrose from source to sink [1]. SUTs contain the typical 12 transmembrane (TM) α-helices of the major facilitator superfamily (MFS) proteins [2–4]. Most SUTs are characterized as energy-dependent proton sucrose/H^+^ symporters that utilize the proton motive force across the membrane to transport sucrose against its concentration gradient [5, 6].

The first *SUT* gene was isolated from spinach by functional complementation in yeast [7]. Later, integrative whole-genome sequence analysis reveals that *SUTs* belong to a small gene family, with nine and five members in *Arabidopsis thaliana* [8] and *Oryza sativa* [9], respectively. Phylogenetic analysis showed that the SUT family could be divided into three subfamilies, SUTI, SUTII, and SUTIII, with the SUTI subfamily present exclusively in eudicots [1, 10–12]. The SUTII subfamily are further divided into two clades SUTIIA and SUTIIB, with the latter being monocot specific [11, 12]. SUTIIA represents an ancestral form of the SUTII subfamily in angiosperms and has a longer central cytoplasmic loop than SUTIIB [12, 13]. The SUTI subfamily members are exclusively located in the plasma membrane and are responsible for loading sucrose into the phloem or for uptake of sucrose into cells of sink tissues [14–21]. While most SUTII members are located in the plasma membrane where they function as sugar sensors, recent studies have shown that MdSUT2.2 reside in the vacuolar membrane, and its overexpression leads to increased sucrose accumulation in the vacuole [22, 23]. Members of the SUTIII subfamily have previously been associated with vacuolar membrane where they are involved in sucrose efflux from the vacuole into the cytosol [24–29]. However, some SUTIII members, such as *Arabidopsis* AtSUT4 [30], barley HvSUT2 [31], *Lotus japonicus* LiSUT4 [25], tobacco NtSUT4 and potato StSUT4 [32], and apple MdSUT1 [33], are located in the plasma membrane. It is worth noting that monocots and eudicots utilize different types of SUTs to load sucrose into the phloem. The SUT1 subfamily that has the highest affinity towards the substrate sucrose is crucial for efficient phloem loading of sucrose in eudicots, whilst monocots utilize the high affinity SUT2 transporters [14, 17, 34, 35]. In addition, SUT4 has a lower affinity for sucrose relative to SUT1 and SUT2 [30].

In addition to their association with sucrose transport, *SUT* genes also play essential roles in a variety of developmental and physiological processes in plants. For example, the *SUT1* gene subfamily participates in regulation of vegetative growth [17], pollen development [36, 37], flowering date [38], fruit and seed development [39, 40], anthocyanin accumulation [41] and tuber yield [42]. The *SUT2* gene subfamily was initially described as sugar sensor [13, 43]. However, this putative sucrose-sensing function was subsequently questioned [2], and increasing evidence shows that *SUT2* genes function as sugar transporter and contribute to grain yield enhancement [44], reproductive organ development [31] and resistance to abiotic stress [23].

Sugar accumulation is strongly associated with fruit yield and quality. Since sugar transporter genes play important roles in sugar accumulation [45, 46], increasing attention has been paid to investigate *SUT* gene family over the past two decades in a wide range of fruit species, such as grapevine [47–49], sweet orange [50], peach [29, 51], pear [52] and apple [53]. A *SUT2* gene, i.e. *MdSUT2.2* in apple, has been recently proven to regulate fruit sugar accumulation in apple [22, 23, 54]. Several *SUT4* genes, including citrus *CsSUT4* [50], grapevine *VvSUC11* [55], apple *MdSUT1* [33] and jujube *ZjSUT4* [56], were found to be potentially associated with fruit sugar accumulation. However, the role of *SUT4* gene family in fruit sugar accumulation still remains elusive.

Apple (*Malus x domestica* Borkh.), a member of the family Rosaceae, is an economically important fruit crop worldwide. It is a diploid but has an autopolyploid origin, with a basic chromosome number of x = 17 and a haploid genome size of approximately 750 Mb [57]. Sugar content is an essential component of fruit organoleptic quality, and thus is given a high priority in apple breeding programs. However, sugar content is inherited quantitatively and few genes responsible for fruit sugar accumulation have been identified although genetic mapping of quantitative trait loci (QTL) for fruit sugar content has been extensively conducted in apple [58–62]. To facilitate understanding of the complex mechanism controlling sugar accumulation in fruit, we investigated the roles of the *SUT* gene family in determining sugar content of apple fruit and an *MdSUT4* gene was found to participate in regulation of fruit sugar accumulation. This study will assist us in understanding of the impact of SUT4 proteins on fruit sugar accumulation. The *SUT4* gene along with its molecular tag can also serve as molecular tools that are useful for genetic improvement of fruit sweetness in breeding programs of apple.

## Results

### Identification of the *SUT* gene family in the apple genome

**Fig. 1:**
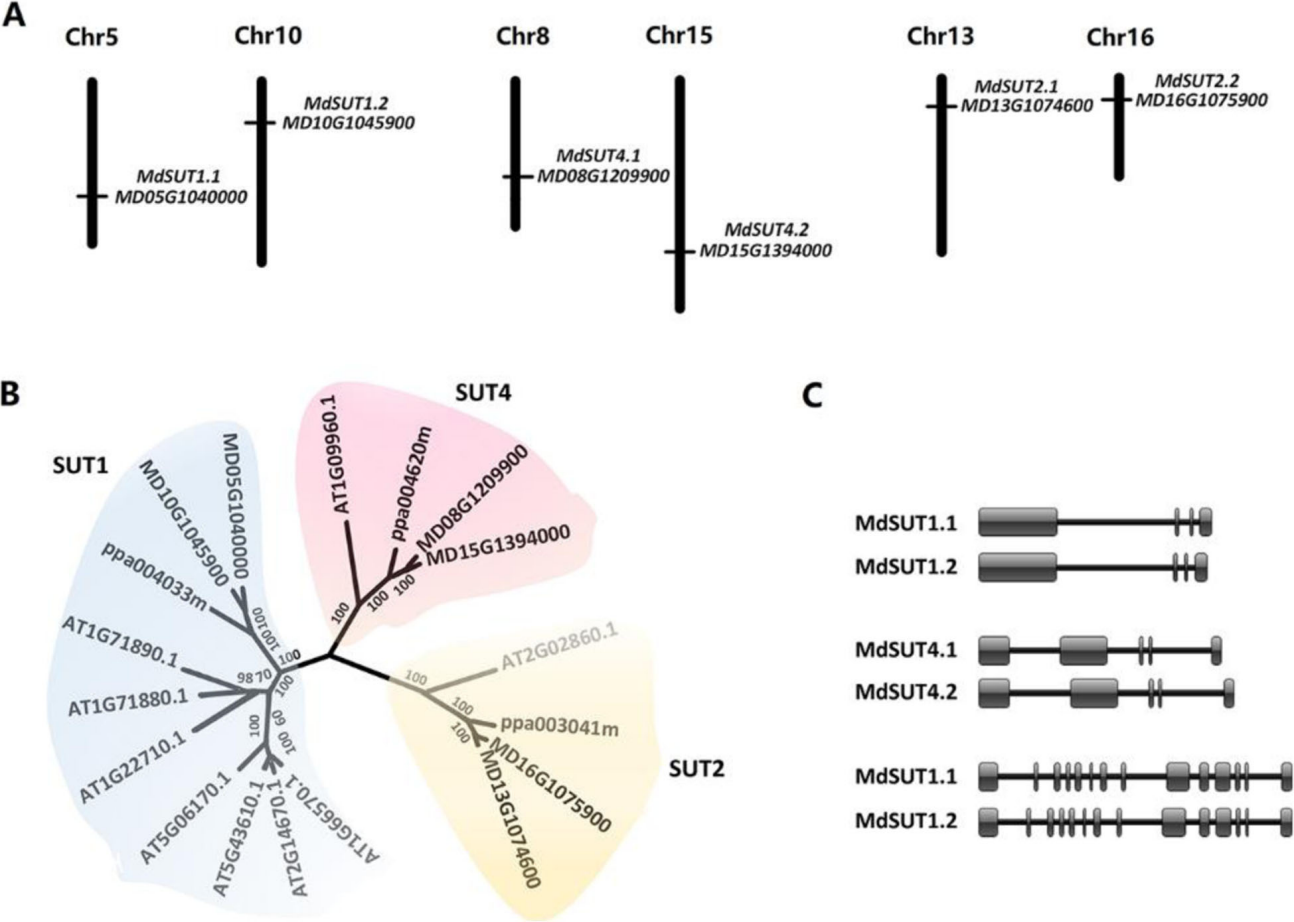
Genes encoding sucrose transporter (SUT) in the apple genome. **a** Chromosomal location of the apple *MdSUTs* and their accession numbers in the Genome Database for Rosaceae (GDR). **b** Phylogenetic tree derived from amino acid sequences of SUTs in apple, peach and *Arabidopsis*. Numbers near branches indicate bootstrap test result with 1000 replicate analyses. C, Genomic structure of the *MdSUT* genes

Screening the apple reference genome sequences of the ‘Golden Delicious’ doubled-haploid GDDH13 revealed a total of six *MdSUT* genes, which are located on different chromosomes (Fig. 1a). The deduced proteins of *MdSUTs* all contained 12 putative transmembrane α-helices (Fig. S1). Phylogenetic analysis showed that the six *MdSUTs* could be classified into three subfamilies, with each containing two members (Fig. 1b), and the *MdSUT* genes were subsequently named according to the standard gene nomenclature [1]. Interestingly, two *MdSUT* genes of the same subfamily shared similar genomic structure and were located on a pair of homologous chromosomes (Fig. 1a, c). The *MdSUT1*, *MdSUT2*, and *MdSUT4* genes contained four, fourteen and five exons, and were located on three homologous pairs of chromosomes, 5–10, 13–16 and 8–15, respectively. In addition, the MdSUT2 proteins had a large central loop between TM6 and TM7, with a length of over 90 amino acids, whereas, the central loop length was less than 45 amino acids for both MdSUT1 and MdSUT4 (Fig. S1). Likewise, the length of N-terminal domain of MdSUT2 was ~ 2.7 times longer than those of MdSUT1 and MdSUT4. These results are consistent with the previous report that the SUT2 subfamily has the extended N-terminus and central loop [13].

### Association between *MdSUTs* and sugar accumulation in apple fruit

**Fig. 2:**
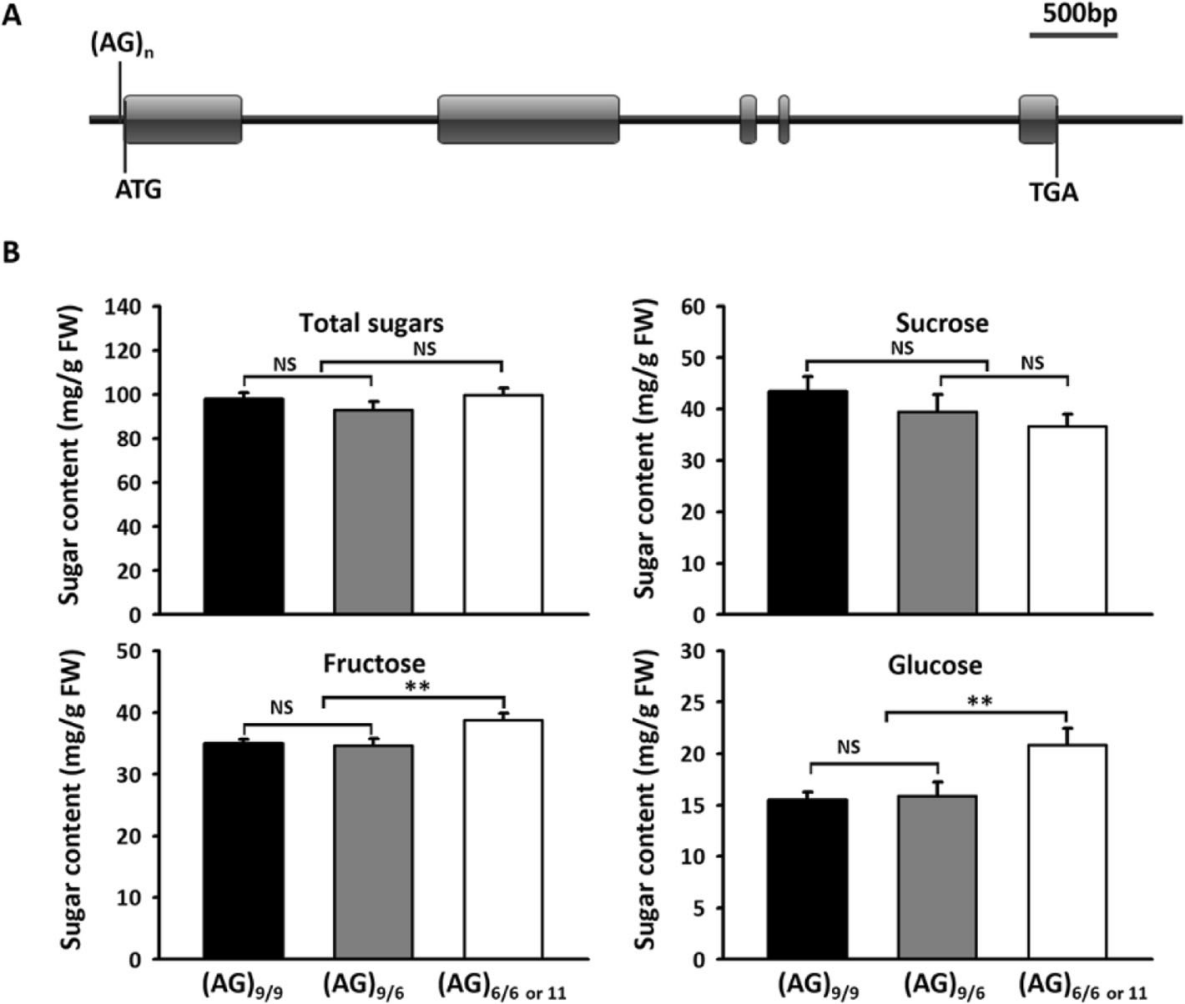
The (AG)_n_ motif site (**a**) and the mean values of sugar contents in apple mature fruits of three different genotypes at the *MdSUT4.1* locus among tested cultivars (**b**). Statistical significance was analyzed by Student’s t-test. **, *P* < 0.01; NS, no significant difference (*P* > 0.05). Error bars represent standard deviation of each genotype data set

Simple sequence repeat (SSR) markers were developed for *MdSUT1.1* and *MdSUT2.1* based on two intronic microsatellites, (CAAAAA)_n_ and (TA)_n_, respectively (Table 1). Two microsatellites located upstream of the start codon, (CT)_n_ and (AG)_n_, were successfully used to develop SSR markers for *MdSUT1.2* and *MdSUT4.1*, respectively. Whereas, SSR markers for *MdSUT4.2* and *MdSUT2.2* were developed based on (TA)_n_ and (CT)_n_ microsatellites, respectively, located downstream of the stop codon. To estimate the association of the *SUT* genes with fruit sugar content, these newly developed SSR markers were used to genotype the 353 apple accessions reported in our previous study [63]. As a result, four polymorphic bands were detected for both *MdSUT1.2* and *MdSUT2.2*, whereas, three, three, two and two polymorphic bands were identified for *MdSUT1.1*, *MdSUT4.1*, *MdSUT4.2* and *MdSUT2.1*, respectively (Fig. S2a, b). Finally, association of the polymorphic loci of *MdSUT* genes with fruit sugar content was analyzed using the candidate gene-based association strategy. The results showed only the (AG)_n_ microsatellite located 15 bp upstream of the start codon of *MdSUT4.1* was significantly associated with fructose content, with the probability values (*P*-value) of 0.0028 (Table 1). Three alleles, (AG)_6_, (AG)_9_, and (AG)_11_, were identified at the *MdSUT4.1* locus by direct sequencing of PCR products (Fig. 2a). Based on the present or absence of the (AG)_9_ on the *MdSUT4.1* locus, all the accessions were divided into three genotypes: (AG)_9/9_, (AG)_6/9_, and (AG)_6/6 or 11_. Accessions with one or two (AG)_9_ alleles had significantly lower fructose content than those with no (AG)_9_ allele (Fig. 2b). However, the (AG)_9_ allele showed no significant impact on either total sugar or sucrose contents.

**Table 1:** SSR markers of *MdSUTs* and their association with soluble sugar content in mature apple fruit

Gene	ID	SSR		*P*-value for association between *MdSUTs* and sugar content			
		Motif^a^	Location	Sucrose	Fructose	Glucose	Total
*MdSUT1.1*	MD05G1040000	(CAAAAA)_3_	1st intron	0.6707	0.9149	0.4246	0.9786
*MdSUT1.2*	MD10G1045900	(CT)_16_	101 bp of USC	0.211	0.3025	0.5504	0.7301
*MdSUT4.1*	MD08G1209900	(AG)_9_	15 bp of USC	0.4133	**0.0028**	0.009	0.0521
*MdSUT4.2*	MD15G1394000	(TA)_20_	17,429 bp of DSC	0.6866	0.478	0.5024	0.845
*MdSUT2.1*	MD13G1074600	(TA)_8_	13th intron	0.8022	0.788	0.4033	0.7082
*MdSUT2.2*	MD16G1075900	(CT)_23_	6395 bp of DSC	0.2024	0.9875	0.2595	0.6202

USC and DSC represent upstream of start codon and downstream of stop codon, respectively. ^a^ The microsatellites were retrieved from the reference genome of GDDH13. *P*-values less than 0.01 are highlighted in black bold

**Fig. 3:**
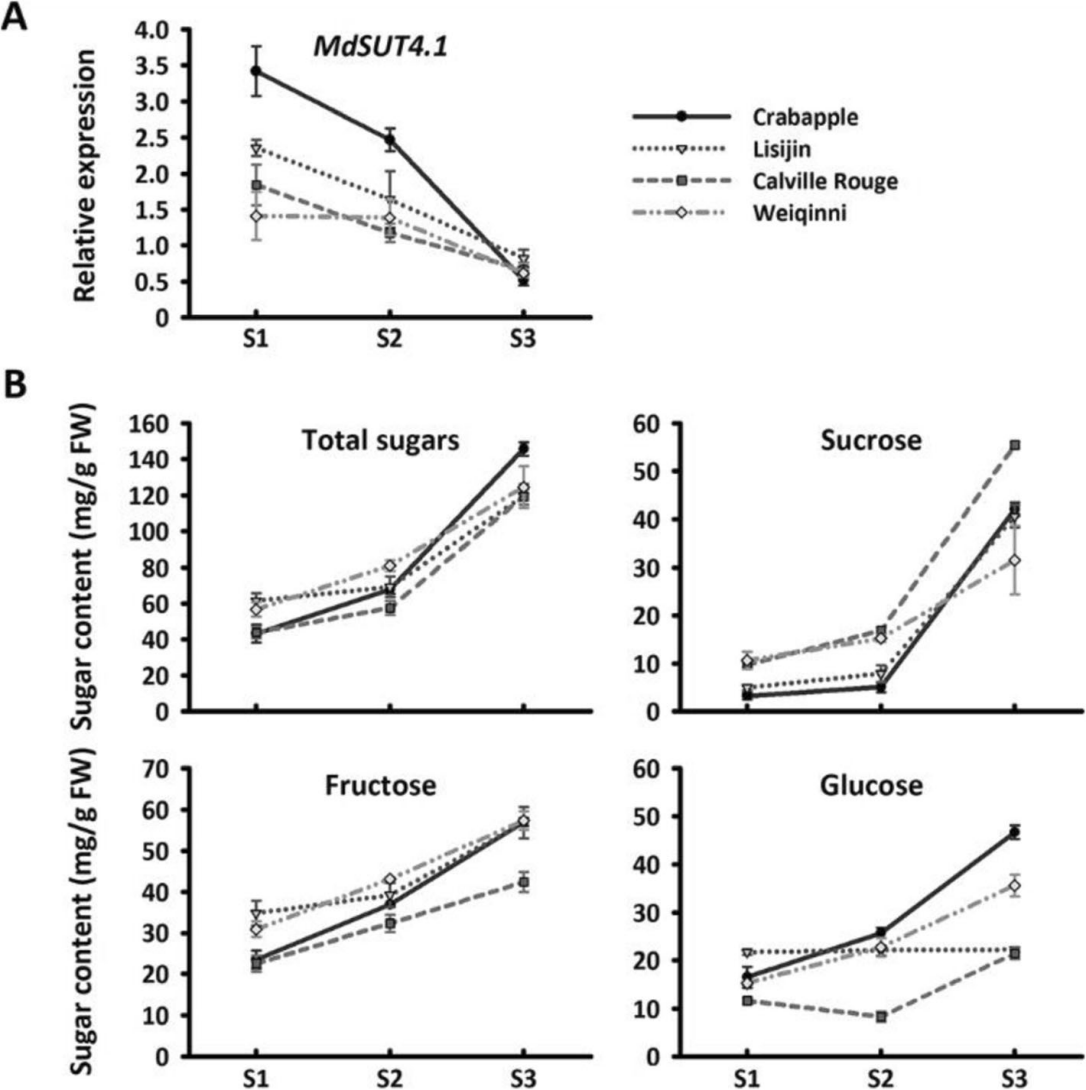
Expression profile of the *MdSUT4.1* gene (**a**) and sugar accumulation (**b**) in fruits of three apple cultivars and one crabapple at various developmental stages. S1-S3 indicate young (60 DAF), expanding (90 DAF) and mature stages (120 DAF), respectively. Error bars represent standard error (SE) of three independent biological replicates

To further confirm the association of the *MdSUT4.1* gene with apple fruit sugar accumulation, we investigated the expression profile of *MdSUT4.1* in fruits of **three** cultivars and one crabapple at different stages of development (Fig. 3). Overall, the *MdSUT4.1* gene was expressed most highly at the young stage, and then showed a decrease in expression throughout fruit development, with approximately 7- to 9-fold decrease at the mature stage. By contrast, sugar accumulation showed a positive trend throughout fruit development, with a peak at the mature stage (Fig. 3). In addition, the expression level of *MdSUT4.1* was significantly negatively correlated with glucose content (*r* = − 0.359, *P* < 0.05), fructose content (*r* = − 0.618, *P* < 0.001), sucrose content (*r* = − 0.604, *P* < 0.001), and total sugar content (*r* = − 0.624, *P* < 0.001). Taken together, these results suggested that *MdSUT4.1* was a candidate involved in fruit sugar accumulation and further subjected to functional analysis.

### Subcellular localization of MdSUT4.1 in tobacco

**Fig. 4:**
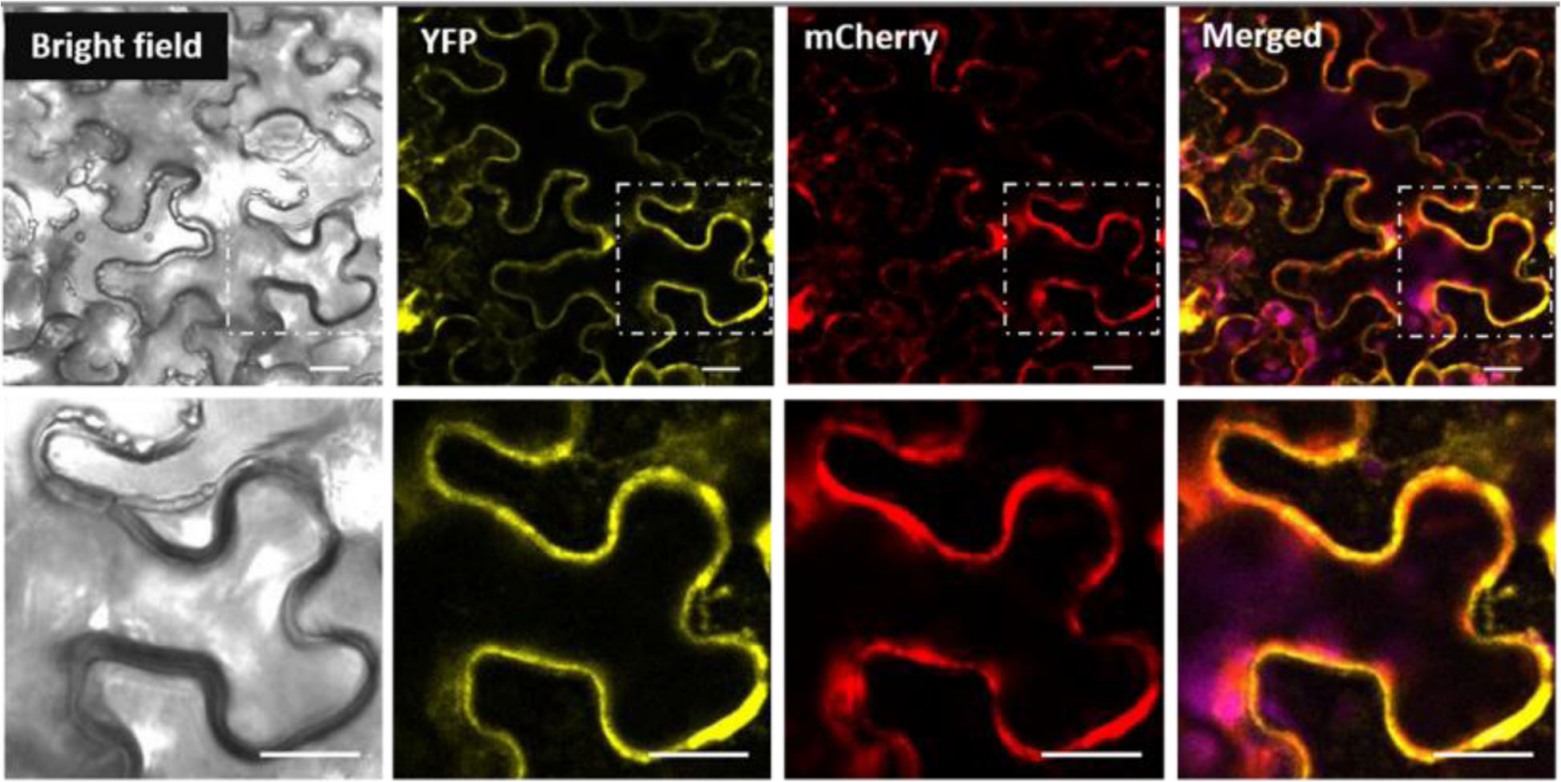
The subcellular localization of MdSUT4.1 in tobacco leaves. Co-expression of pFGC-eYFP-MdSUT4.1 and tonoplast marker vac-rk CD3–975 in bright field, YFP channel, mCherry channel and merged channel, respectively. Bottom is the enlarged images indicated in an upper lane. Scale bars: 20 μm

As mentioned above, MdSUT4.1 contained twelve putative transmembrane domains. To further determine subcellular localization of MdSUT4.1, the yellow fluorescent protein (YFP) was fused to the C-terminus of MdSUT4.1. The MdSUT4.1-YFP fusion construct driven by CaMV 35S promoter was transiently expressed in tobacco (*Nicotiana benthamiana*), and the MdSUT4.1-YFP fusion protein was found to reside in the tonoplast (Fig. 4). In addition, we did co-localization with the standard vacuolar membrane marker Vac-rk CD3–975 to confirm the subcellular location of MdSUT4.1 (Fig. 4). Laser-scanning confocal fluorescence microscopy revealed that the mCherry fluorescence from the tonoplast marker Vac-rk CD3–975 was merged with the YPF fluorescence from MdSUT4.1-YFP. This result indicated that MdSUT4.1 is a tonoplast localized protein.

### Overexpression of the *MdSUT4.1* gene in strawberry and apple callus

**Fig. 5:**
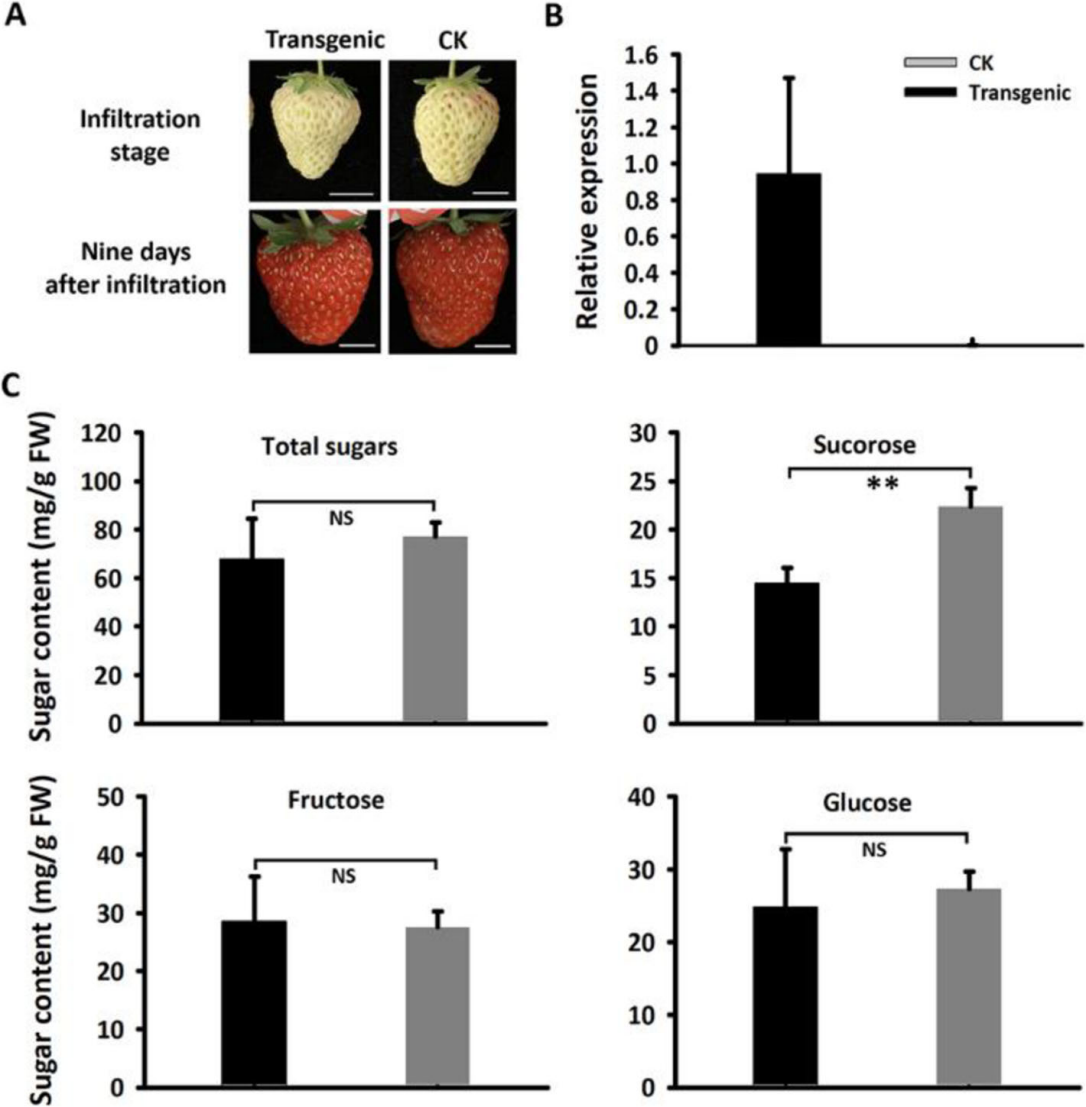
Overexpression of *MdSUT4.1* in strawberry fruit. **a** Strawberry fruits infiltrated individually with *MdSUT4.1* (transgenic) and the entry vector pSAK277 (CK). Scale bar, 1 cm. **b** Relative expression of *MdSUT4.1* in strawberry fruits infiltrated with *MdSUT4.1* and the entry vector, respectively. **c** Sugar accumulation in strawberry fruits infiltrated with *MdSUT4.1* and the entry vector, respectively. Statistical significance was analyzed by Student’s t-test. **, *P* < 0.01; NS, no significant difference (*P* > 0.05). Error bars represent SE of three biological replicates

**Fig. 6:**
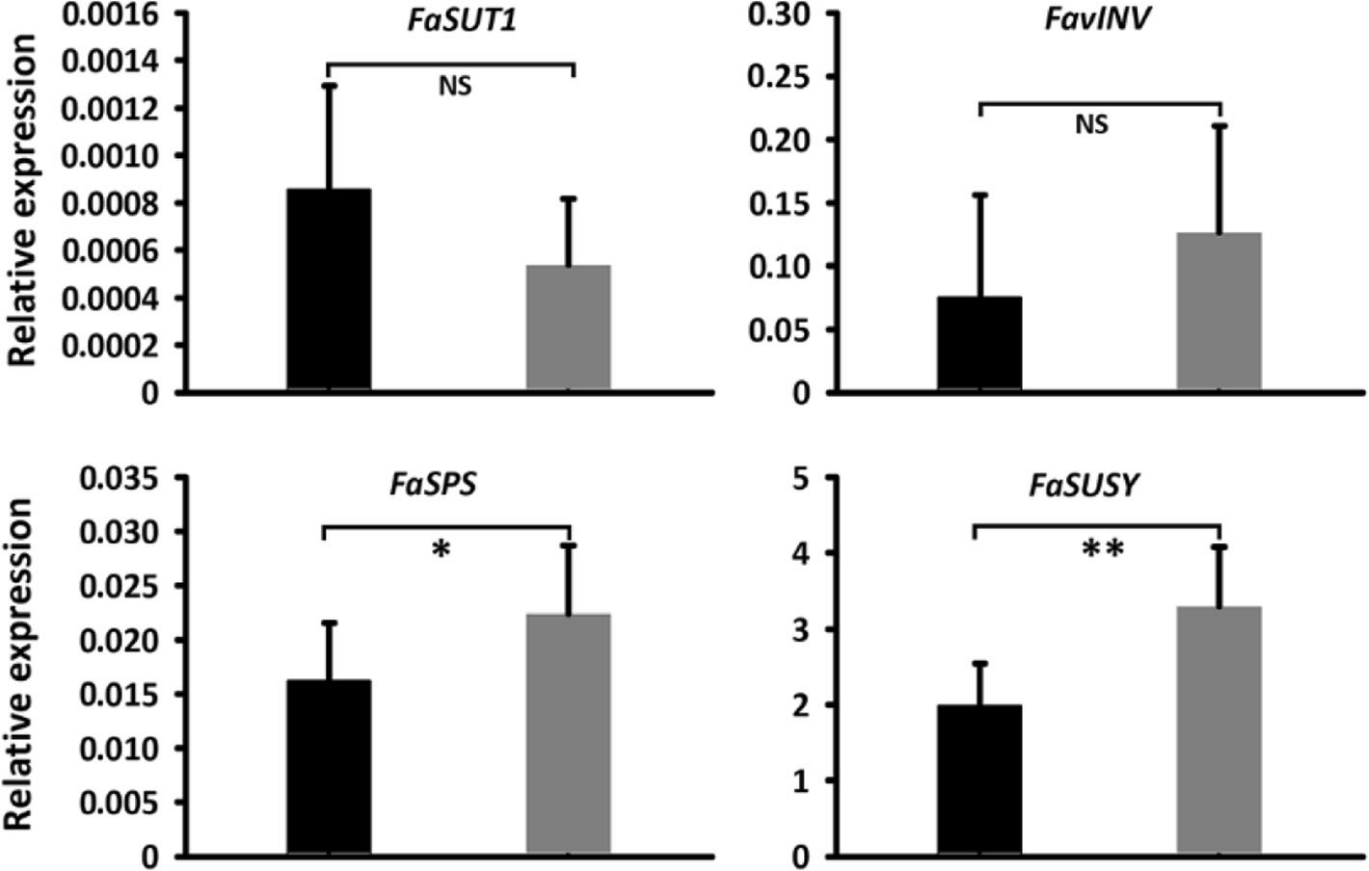
Expression of sugar metabolism and sugar transporter genes in strawberry fruits infiltrated with *MdSUT4.1* (black column) and the entry vector pSAK277 (control, gray column). Statistical significance of differences between mean values was analyzed by Student’s t-test. **, *P* < 0.01; *, *P* < 0.05; NS, no significant difference (*P* > 0.05). Error bars represent standard error (SE) of three biological replicates. SPS, sucrose phosphate synthase; SUSY, sucrose synthase; vAINV, vacuolar acid invertase

To examine whether MdSUT4.1 was able to transport sugars, it was transiently transferred into immature white fruit of strawberry (Fig. 5a). Quantitative real time PCR (qRT-PCR) analysis showed that *MdSUT4.1* was highly expressed in transgenic fruit (Fig. 5b). Sucrose content decreased significantly by 35% in fruit overexpressing *MdSUT4.1* compared to fruit infiltrated with empty vector (Fig. 5c). However, the glucose, fructose, and total sugar contents showed no significant difference between fruits overexpressing *MdSUT4.1* or introducing empty vector. qRT-PCR was conducted to investigate expression levels of genes related to sucrose metabolism or transport, including *FaSPS* (sucrose phosphate synthase), *FaSUSY* (sucrose synthase), *FavAINV* (vacuolar acid invertase) and *FaSUT1*, which are proven to play important roles in determining fruit sucrose content in strawberry [40, 64]. As a result, expression levels of *FaSPS* and *FaSUSY* were significantly lower in fruits overexpressing *MdSUT4.1* than in fruits infiltrated with empty vector, but no significant difference in expression level was observed for *FaSUT1* and *FavINV* (Fig. 6). This was consistent with the result that sucrose accumulation was decreased in fruits overexpressing *MdSUT4.1*.

**Fig. 7:**
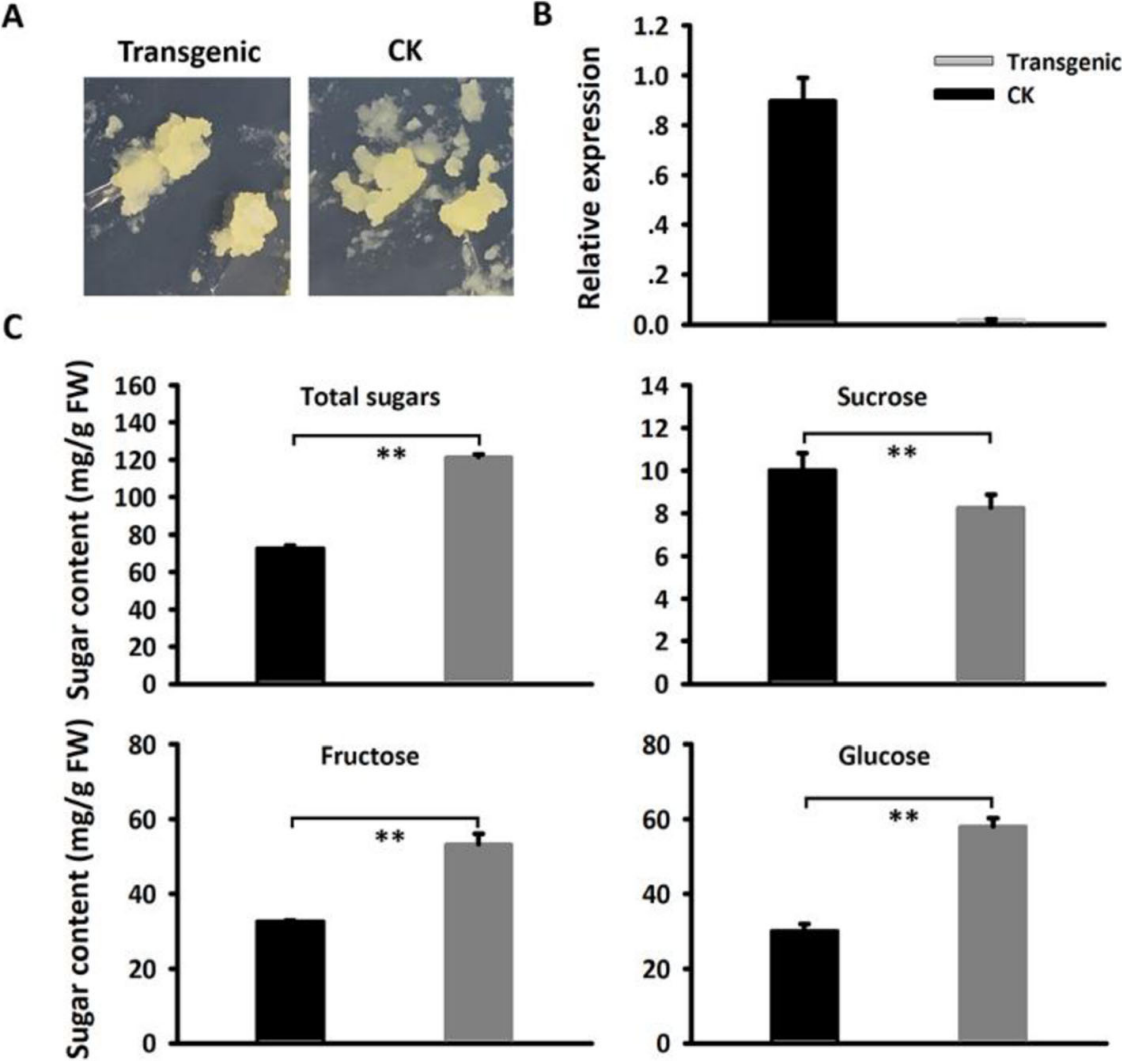
Overexpression of *MdSUT4.1* in apple calli. **a** Apple calli infiltrated with *MdSUT4.1* (transgenic) and the entry vector pSAK277 (CK), respectively. **b** Expression of *MdSUT4.1* in transgenic calli and CK. **c** Sugar accumulation in transgenic calli and CK. Statistical significance was analyzed by Student’s t-test. **, *P* < 0.01. Error bars represent SE of three biological replicates

**Fig. 8:**
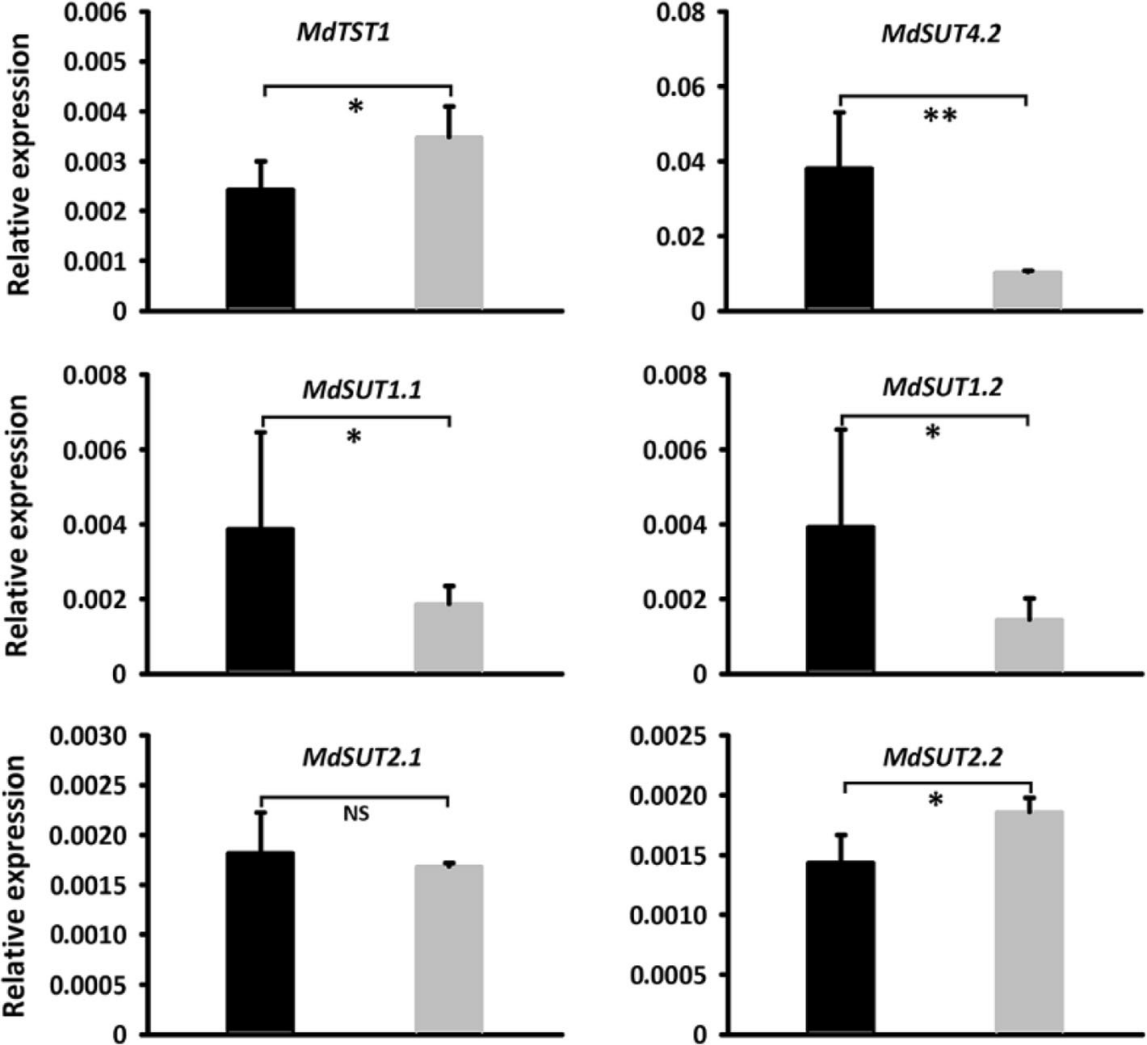
Expression of *MdTST1* and *MdSUTs* in apple calli infiltrated with *MdSUT4.1* (black column) and the entry vector pSAK277 (control, gray column). Statistical significance was analyzed by Student’s t-test. Asterisk **, *P* < 0.01; *, *P* < 0.05; NS, no significant difference (*P* > 0.05). Error bars represent standard error (SE) of three biological replicates

**Fig. 9:**
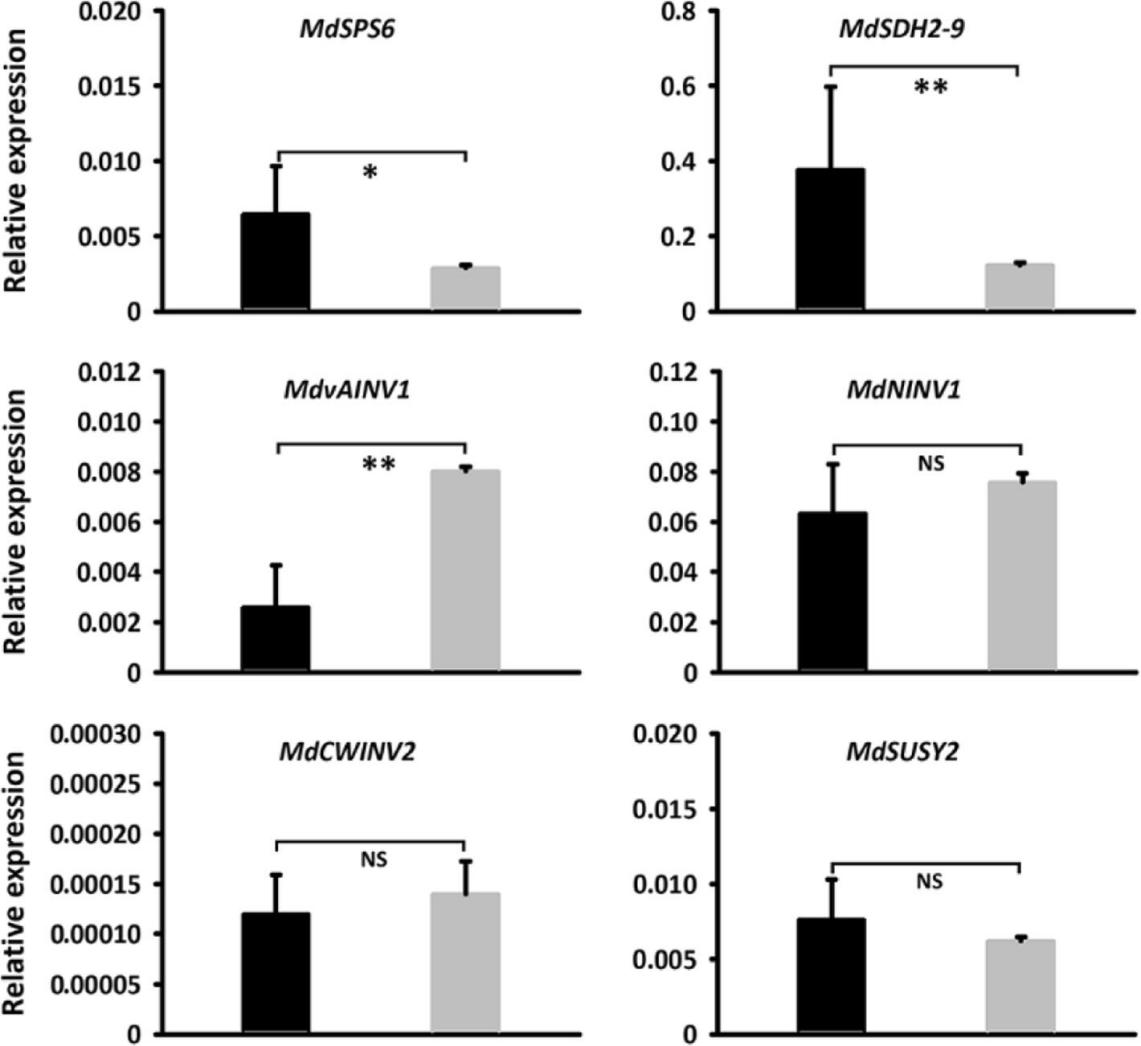
Relative expression level of sugar metabolic gene in apple calli infiltrated with *MdSUT4.1* (black column) and the entry vector pSAK277 (control, gray column). Statistical significance was analyzed by Student’s t-test. **, *P* < 0.01; *, *P* < 0.05; NS, no significant difference (*P* > 0.05). Error bars represent SE of three biological replicates. SPS, sucrose phosphate synthase; SDH, sorbitol dehydrogenase; vAINV, vacuolar acid invertase; NINV, neutral/cytosol invertase; CWINV, cell wall invertase; SUSY, sucrose synthase

To further confirm the function of the *MdSUT4.1* gene involved in fruit sugar accumulation, it was also transferred into the apple callus (Fig. 7a). qRT-PCR assay showed that the *MdSUT4.1* gene was highly expressed in transgenic lines (Fig. 7b). The concentrations of sucrose in calli overexpressing *MdSUT4.1* increased significantly by 1.77 mg/g FW, whilst the concentrations of glucose, fructose, and total sugars dramatically dropped by 27.96, 20.68, and 48.88 mg/g FW, respectively, compared to calli introducing the empty vector (Fig. 7c). qRT-PCR analysis was conducted to investigate expression levels of sugar transport-related genes (Fig. 8). As a result, a *tonoplast sugar transporter MdTST1* and *MdSUT2.2* showed significantly lower levels of expression in calli overexpressing *MdSUT4.1* than in calli introducing the empty vector, but the opposite trend was observed for *MdSUT4.2*, *MdSUT1.1* and *MdSUT1.2*. By contrast, *MdSUT2.1* showed no significant difference in expression level between calli overexpressing *MdSUT4.1* and calli introducing the empty vector. Moreover, expression levels of sugar metabolism-related genes that were reported to affect fruit sugar accumulation in apple [65] were also investigated (Fig. 9). *MdvAINV1* showed significantly lower levels of expression in calli overexpressing *MdSUT4.1* than in calli introducing the empty vector, while the opposite trend was observed for *MdSPS* and *MdSDH2–9* (sorbitol dehydrogenase) genes. By contrast, *MdNINV1* (Neutral/cytosol invertase), *MdSUSY2*, and *MdCWINV2* (cell wall invertase) showed no significant difference in expression level between calli overexpressing *MdSUT4.1* and calli introducing the empty vector.

## Discussion

### The complexity of mechanisms underlying the targeting of SUT4 proteins to the tonoplast

In fruit, soluble sugars are mainly stored in the cell vacuole, and their uptake into or release out of the vacuole is catalyzed by tonoplast-localized transporters. A di-leucine motif (LXXLL) within the N- and/or C-terminal domains is responsible for the targeting of sugar transporters to the tonoplast in plants [11, 66, 67]. A variety of SUT4 subfamily members that contain the di-leucine motif, such as *Arabidopsis* AtSUT4 (also known as AtSUC4) [24], *Lotus japonicas* LjSUT4 [25], barley HvSUT2 [24], rice OsSUT2 [68] and *Populus* PtaSUT4 [26], have been shown to reside in the vacuolar membrane. In fruit trees, PpSUT4 in peach, MdSUT4.1 and MdSUT4.2 in apple and PbSUT2 in pear all contain the vacuolar targeting di-leucine motif LXXLL (Fig. S3), thus, they are expected to localize in the tonoplast. MdSUT4.2 is also known as MdSUT1 in a previous report [33]. Like MdSUT4.1, PpSUT4 was shown to reside in the vacuolar membrane by its transient expression in tobacco leaf [29]. However, both MdSUT1 and PbSUT2 were shown to localize in the plasma membrane via their ectopic expression in *Arabidopsis* protoplasts and onion epidermis cells [33, 52]. This inconsistency indicates that other unknown factors, besides the di-leucine motif, may also influence subcellular localization of SUT4 subfamily members.

### MdSUT4.1 participates in regulation of apple fruit sugar accumulation

Our study showed that *MdSUT4.1* was significantly associated with fruit sugar accumulation in apple and is located on an interval of the sweetness QTL on LG8 [58]. The expression levels of *MdSUT4.1* displayed a negative correlation with fruit sugar accumulation, and its overexpression in strawberry and apple callus showed an overall negative impact on sugar accumulation. These results demonstrate that the *MdSUT4.1* gene is a strong candidate for the regulation of fruit sugar accumulation in apple.

As mentioned above, MdSUT4.1 resides in the vacuolar membrane, and its transient overexpression in strawberry significantly decreased sucrose accumulation in fruit. This suggests that MdSUT4.1 has similar functionality as *Arabidopsis* AtSUT4 and maize ZmSUT2, both of which are SUT4 subfamily members and function to remobilize sucrose out of the vacuole [27, 28, 69]. This hypothesis is consistent with the finding that the expression of sucrose synthesis gene *FaSPS* showed a significant decrease in strawberry fruit overexpressing MdSUT4.1 compared with fruit infiltrated with the empty vector. This change in expression level of *FaSPS* may be attributed to feedback from the efflux of sucrose from the vacuole caused by overexpression of *MdSUT4.1*.

Similarly, the overexpression of *MdSUT4.1* in apple callus resulted in a significant decrease in hexose accumulation, which is consistent with the finding that the expression of *MdSUT4.1* shows a negative correlation with sugar accumulation in apple fruit. MdSUT4.1 probably also mediates the efflux of sucrose from the vacuole in apple calli as this hypothesis is well consistent with the result that the expression of *MdvAINV1* responsible for sucrose cleavage in the vacuole significantly decreased in apple calli overexpressing *MdSUT4.1*. The slight increase of sucrose accumulation in apple calli overexpressing *MdSUT4.1* may be due to activation of *MdSUT1.1* and *MdSUT1.2* as the *SUT1* genes are known to serve as the high-affinity sucrose transporters and function in sucrose uptake into sink storage tissues [70, 71].

Previous studies have demonstrated that SUT4 can interact with other sugar transporters such as SUT1 and TST [29, 43, 72]. Thus, it cannot be excluded that overexpression of *MdSUT4.1* in apple callus may affect its interaction with other sugar transporter such as MdTST, resulting in the change in sugar accumulation. In addition, our results show that overexpression of *MdSUT4.1* results in a cascade response of sugar metabolism and transporter genes to affect sugar accumulation, which is similar to previous reports [32, 73].

In apple, sorbitol is the predominant sugar in the phloem, but it only accounts for about 5% in mature fruit as most is converted to fructose [74]. The expression of genes encoding sorbitol transporter (SOT), including *MdSOT1*, *MdSOT4* and *MdSOT5.3*, decreased in apple calli overexpressing *MdSUT4.1* (Fig. S4). Thus, it is worthy of further study to ascertain whether overexpression of *MdSUT4.1* has an impact on expression of *MdSOTs*, resulting in a change in sugar accumulation in apple fruit.

## Conclusions

Collectively, our results show that the tonoplast-localized sucrose transporter, MdSUT4.1, participates in regulating fruit sugar accumulation in apple. Our results are useful for better understanding of the impact of SUT4 proteins on fruit sugar accumulation. In addition, the molecular marker of *MdSUT4.1* may serve as a tool for genetic improvement of fruit sweetness in breeding programs of apple.

## Methods

### Plant material

All apple accessions used in this study are maintained at Xingcheng Institute of Pomology, the Chinese Academy of Agricultural Sciences, Xingcheng, Liaoning province, China. Young leaves were collected for each cultivar, frozen in liquid nitrogen, and then stored at − 80 °C until use. Fruit samples for sugar content measurement were randomly harvested at mature stage in 2015, which was estimated based on previous records of maturity date, skin background color and blush development, and seed color changing to brown. Moreover, one crab apple (*Malus coronaria*) and three cultivars, ‘Calville Rouge’, ‘Lisijin’, and ‘Weiqinni’, were selected for qRT-PCR analysis. Fruit samples were harvested at young, immature and mature stages, which corresponded to 60, 90, and 120 days after flowering (DAF), respectively. Three biological replicates with 10 fruits were conducted for each accession. Fruit samples were frozen in liquid nitrogen and stored either at − 80 °C for qRT-PCR analysis or at − 40 °C for sugar measurement. In addition, strawberry cultivar ‘Hongyan’ was kindly provided by Institute of Economic Crops, Hubei Academy of Agricultural Sciences, Wuhan, China and it was grown in the greenhouse with natural light and temperature of 10 °C – 26 °C. Apple calli induced from flesh of apple cultivar ‘Orin’ was kindly provided by Prof. Qingmei Guan at Northwest Agriculture University, Yangling, China.

### Measurement of sugar contents in fruits

Sugar content was measured by high performance liquid chromatography (HPLC) according to our previously reported protocol [75]. Total sugars in this study represents the sum of dominant soluble sugars, including sucrose, fructose and glucose, which together account for over 95% of the total sugar in apple fruits [74]. Briefly, fruit samples were ground into powder in liquid nitrogen using an A11 Basic Analytical Mill (IKA-Werke, Staufen, Germany) and approximately 0.5 g of powder was transferred to a clean Eppendorf tube. Soluble sugars were extracted with 6 mL deionized water by ultrasonic treatment for 15 min, and then centrifuged at 5000×g for 15 min. The resultant supernatant was filtered through a 0.22 μm Sep-Pak filter (ANPEL, China). The filters were subjected to sugar content measurement using HPLC system (Agilent 1260 Infinity, Agilent, Waldbronn, Germany) with refractive index detector (RID). The injection volume was 20 μL, and chromatographic separation was performed using a CarboSep CHO-620 Ca column (300 × 6.5 mm, 10 μm particle size) and CarboSep CHO-620 Cartridge (Transgenomic, San Jose, CA, USA). The mobile phase was deionized water at the flow rate of 0.5 mL/min and the column temperature was maintained at 90 °C. Sugar concentration was estimated by comparison with the values from a standard curve. Sucrose (CAS number 57–50-1), glucose (CAS number 50–99-7), and fructose (CAS number 57–48-7) standards were purchased from Sigma. Each accession had three biological replicates.

### RNA isolation and qRT-PCR analysis

Total RNA was extracted with the RNAprep pure Plant kit (Tiangen, Beijing, China) following the manufacturer’s instructions, and genomic DNA contamination was removed using DNase I (Takara, Dalian, China). The synthesis of cDNA templates was conducted using TransScript One-Step gDNA Removal and cDNA Synthesis SuperMix (TRANS, Beijing, China) following the manufacturer’s instructions. The qRT-PCR assay was carried out with TB Green® Premix Ex Taq™ II (Tli RNase H Plus) (Takara, Dalian, China), and amplifications were performed on Applied Biosystems Step One Plus Real-Time PCR Systems (Life Technologies Corporation, Carlsbad, CA, USA). Melting curve was analyzed at the end of 40 cycles to confirm specific amplification products. A previously reported actin gene [76] was used as a constitutive control, and negative control for each sample was also performed. Each sample had three biological replicates. Primer sequences are listed in Table S1.

### Identification of *SUT* genes in the apple genome and their classification

Identification of *SUT* genes was initially conducted based on the apple genome annotation database for GDDH13 [77], and six *MdSUT* genes were identified. Subsequently, the coding sequence of each *SUT* gene was further compared against the apple draft genome using the online program BlastN (https://www.rosaceae.org/blast), with an *E* value cutoff of 1e-10, and no additional member was identified. To validate the annotation of the *MdSUT* genes, their protein sequences were BLASTed against GenBank’s non-redundant protein (NR) and Swiss-Prot protein databases using BLASTp, an *E* value cutoff of 1e-5. Chromosomal locations of *MdSUTs* were presented according to the draft genome sequence of GDDH13 [77]. Prediction of protein transmembrane domains was conducted using PSIPRED (http://bioinf.cs.ucl.ac.uk/psipred/) [78]. Gene structures were drawn using the visualized software IBS Version 1.0 [79].

To classify *MdSUTs*, their amino-acid sequences were aligned with those of *SUT* genes from *Arabidopsis* and peach using MEGA5 with default parameters. The resulting data matrix was used to construct phylogenetic tree using the Neighbor-Joining method. Bootstrap values were estimated from 1000 replicates.

### Development of gene-tagged markers and candidate gene-based association analysis

Genomic DNA sequences of each *MdSUT* were retrieved from the draft genome of GDDH13 [77] and then subjected to screen SSRs with ≥6 repeat units using microsatellite repeats finder (http://insilico.ehu.eus/mini_tools/microsatellites/). Primers of SSR markers were designed using Primer premier 5. SSR polymorphism was detected using polyacrylamide gel electrophoresis in combination with silver staining following a previous report [80].

Polymorphic SSRs for *MdSUTs* were used to screen the same collection of 353 apple accessions as in our previous study [63], and their primer sequences are listed in Table S2. Association of *MdSUTs* with fruit sugar concentration was carried out with TASSEL version 3.0 using a mixed linear model, with *P* < 0.01 as the criterion for significant marker–trait association. The relative kinship (K matrix) and the most likely K value (Q matrix) were the same in our previous study [63]. The Bonferroni threshold was set at ≤1/n, where n represents the sample size.

### Subcellular localization analysis of MdSUT4.1 in tobacco

YFP fragment was amplified from pEarleyGate104 and then cloned into the BamHI-SpeI sites of pFGC5941 using Hieff CloneTM Plus One Step Cloning Kit (Yeasen, Shanghai, China), resulting in a pFGC-eYFP vector. Amplification of the full-length coding sequences of *MdSUT4.1* was performed using cDNA templates that were prepared from fruits of ‘Calville Rouge’. The purified PCR products were inserted into the BamHI site of the pFGC-eYFP vector to generate a pFGC-eYFP-MdSUT4.1 construct using In-Fusion® HD Cloning Kit (Takara, Dalian, China) following the manufacturer’s instructions. Primer sequences used for expression vector construction are listed in Table S3.

The vacuolar membrane marker vac-rk CD3–975 [81] and the pFGC-eYFP-MdSUT4 construct were individually transferred into *Agrobacterium tumefaciens* strain GV3101 (pMP90) using the heat shock method, spread onto agar plates, and incubated at 28 °C for 3 days. A single colony was picked, suspended in LB medium, and incubated at 28 °C overnight under shaking. Agrobacterium cells were collected by centrifugation at 4000 g for 5 min and resuspended in infiltration buffer (10 mM MES, 10 mM MgCl_2_, 450 μM acetosyringone, pH 5.6). Leaves of 4–5 weeks old *N. benthamiana* were infiltrated with bacterial cultures and the fluorescence was detected two days after infiltration using the confocal microscope (TCS SP8, Leica, Microsystems, Wetzlar, Germany). All scanners used a × 20 objective lens to acquire the digital images. A 512 nm laser was used for the excitation of YFP, while mCherry was excited by 552 nm laser. Fluorescence emission was detected in the ranges of 530–550 nm (YFP) and 600–650 nm (mCherry), respectively.

### Functional analysis of *MdSUT4.1* in strawberry and transgenic apple callus

The entire CDS of *MdSUT4*.*1* was amplified and inserted into the site of the expression vector pSAK277 between EcoRI and XbaI enzyme sites via homologous recombination. Primer sequences are listed in Table S3. The recombinant vector pSAK277-MdSUT4.1 and the empty vector pSAK277 were individually transferred into *A. tumefaciens* strain EHA105 with the heat shock method, and then spread onto agar plates containing 50 mg/L streptomycin, 50 mg/L gentamycin and 100 mg/L spectinomycin. After incubation at 28 °C for 3 days, single colony was picked, suspended in LB medium, and incubated at 28 °C overnight with shaking. Agrobacterium cells were collected by centrifugation at 4000 g for 5 min and then used to infiltrate fruit of strawberry cv. Hongyan and apple calli that were induced from flesh of cv. Orin.

Transient overexpression of *MdSUT4*.*1* in strawberry was conducted according to a previous report [82]. Briefly, the collected bacterial pellets were resuspended in infiltration buffer (50 mM MES, 5 mg/mL D-glucose, 2 mM Na_3_PO_4_, 100 μM acetosyringone) and the density of cells was adjusted to an OD _600nm_ of 0.2. The suspension was injected into the flesh of immature white fruits with needle of syringes, and the infiltrated plants were placed in the greenhouse with natural light and temperature of 10 °C–26 °C. Fruit samples were collected 9 days after infiltration and then subjected to qRT-PCR analysis and sugar content measurement.

For apple callus transformation, the collected bacterial pellets were resuspended in 5% sucrose solution (pH 5.3) and the density of cells was adjusted to an OD _600nm_ of 2.0. Apple calli were immerged in the suspension for 15 min, collected by filtration through sterile nylon net (200 mesh), and then cultured on MS medium containing 3% sucrose, 1 mg/L 2,4-D, 1 mg/L 6-BA and 400 mg/L cefalexin for two days at 25 °C under dark condition. Subsequently, the transgenic apple calli were washed 5 times with sterile Mili-Q water containing 400 mg/L cefalexin, dried on filter, and then cultured on MS selection medium containing 3% sucrose, 1 mg/L 2,4-D, 1 mg/L 6-BA, 400 mg/L cefalexin and 50 mg/L Kanamycin. After successive subculture for three callus generations, transgenic calli were subjected to sugar content measurement using HPLC. In addition, transgenic calli were confirmed by PCR amplification and expression of *MdSUT4.1* was quantified using qRT-PCR analysis. Primer sequences are listed in Table S1.

## Supplementary information

**Additional file 1: Table S1.** Sequences of primers used for qRT-PCR analysis.

**Additional file 2: Table S2.** Primer sequences of gene-tagged SSR markers for six *MdSUT* genes in apple.

**Additional file 3: Table S3.** Sequences of primers used for vector construction.

**Additional file 4: Figure S1.** A schematic of the predicted topology of MdSUTs in apple. AA, amino acid.

**Additional file 5:Figure S2.** An example of genotyping of apple cultivars using SSR markers of the *MdSUT* genes.

**Additional file 6: **Figure S3. Alignment of vacuolar targeting di-leucine motif (LXXLL) in the N-terminus of SUT4 subfamily members in apple, peach, pear and *Arabidopsis*.

**Additional file 7: **Figure S4. Expression of *MdSOTs* in apple calli overexpressing *MdSUT4.1* (black column) and introducing entry vector pSAK277 (control, gray column).

## Data Availability

The datasets used and analyzed during the current study available from the corresponding author on reasonable request.

## Abbreviations

SUT: Sucrose transporter
TST: Tonoplast sugar transporter
SOT: Sorbitol transporter
SPS: Sucrose phosphate synthase
SDH: Sorbitol dehydrogenase
vAINV: Vacuolar acid invertase
NINV: Neutral/cytosol invertase
CWINV: Cell wall invertase
SUSY: Sucrose synthase
DAF: Days after flowering
qRT-PCR: Quantitative real time PCR
HPLC: High performance liquid chromatography
