# Supplementary material for: The sucrose transporter MdSUT4.1 participates in the regulation of fruit sugar accumulation in apple
Source: BMC Plant Biol. 2020 May 6;20:191. doi: 10.1186/s12870-020-02406-3 (PMC7203859; doi:10.1186/s12870-020-02406-3)
Supplement: Supplementary file 1 — Additional file 1: Table S1. Sequences of primers used for qRT-PCR analysis. [file 12870_2020_2406_MOESM1_ESM.docx]

**Table S1.** Sequences of primers used for RT-qPCR analysis

| **Species** | **Tissue** | **Gene** | **Primer (5'→ 3')** | | |
| --- | --- | --- | --- | --- | --- |
|  |  |  | Forward | | Reverse |
| Apple | Fruit | *MdSUT4.1* | AGAGAAGGAAGGCATATATTTGAGAG | GAGTCAAGAGCGAGAGCTGTAGG | |
|  |  | *Actin* | TGACCGAATGAGCAAGGAAATTACT | TACTCAGCTTTGGCAATCCACATC | |
|  | Callus | *MdSUT4.1* | TGATTTACCTCCAGTTGGCATTG | GCCAAGTCCCAAAGACTCAATTC | |
|  |  | *MdSUT4.2* | GTCACCATGCCAGCTCCAGAC | GAATTGGATTCCACACGCGAC | |
|  |  | *MdSUT2.1* | CAGATGGTTTCCTTTCTTGTTGAG | TGCTGATACATAGTGAGTGGAACCT | |
|  |  | *MdSUT2.2* | TTCCTTTTGAGTAGAGCTTGCTGTG | CTCAAGACCATGTTGTTGGGGAT | |
|  |  | *MdSUT1.1* | GTGGTAATTTACCGGCATTTGTCG | AAGCTAGAGGCCGTAGGGCAAG | |
|  |  | *MdSU1.2* | CGAAGACAGAAAGTGTGTGTCCTG | GTAGGGGGTGAGGAGGGAGAG | |
|  |  | *MdvAINV1* | AGGTCAAGGCAGGCTCAGTGCT | CCTAAAGCACCACGATGGGAAGC | |
|  |  | *MdNINV1* | GTCCATTGTTTCATCATTGGGTAC | GGTCGCTGCCAGTGATTATACG | |
|  |  | *MdCWINV2* | TTCAAAGCTAAAGGCAGACACG | GTAAATCTACATCTACAAAGCCAGC | |
|  |  | *MdSUSY2* | TGTGGTTGGTGGTTACATGGATG | GCTGCTATCCATCGGAACTGAC | |
|  |  | *MdSPS6* | AGGTTCTGTTGAGTATGGCAGTGAG | GTGCTTCAAGTGCCGCTGAGA | |
|  |  | *MdSDH2-9* | ACACCATCAAGATCCTACCTTTC | CATTTCATGGTCTTGAGGTAGTG | |
|  |  | *MdSOT1* | GCCGTGAATAGGTTGATGAGTG | CTTCAAGGGTTCTGCCTTGTG | |
|  |  | *MdSOT2.2* | GTTGTTCTTGCCATCGGTGTC | ACAATGTCGTCGTTGGACTGTT | |
|  |  | *MdSOT4* | TTGGTTTCCACTTCTTTCAGCA | TGGAAAGTCCAACTCCCACAG | |
|  |  | *MdSOT5.3* | CTTTCTTTACGGGGGAATTGCT | TCTTCTAGGGTTCTACCTTGGGTCT | |
|  |  | *Actin* | TGACCGAATGAGCAAGGAAATTACT | TACTCAGCTTTGGCAATCCACATC | |
| Strawberry | Fruit | *MdSUT4.1* | TGATTTACCTCCAGTTGGCATTG | GCCAAGTCCCAAAGACTCAATTC | |
|  |  | *FavAINV* | CCGATACCGAGTCCGATGA | TTCAACAACTGCTCCTGCTT | |
|  |  | *FaSPS* | GAATGTCCCTATGTTATTTACTGG | TCCTGT0CTGGTGCTGGTTAT | |
|  |  | *FaSUSY* | TTATCCCTCGCATTCTTATT | CAATTCCCTTCTCGGTTCTA | |
|  |  | *FaSUT1* | CTACAGCGACCGTAACACC | ACAACAAATACAGCCACAGC | |
|  |  | *Actin* | GGGCCAGAAAGATGCTTATGTCGG | GGGCAACACGAAGCTCATTGTAGAAG | |
