# Supplementary material for: The sucrose transporter MdSUT4.1 participates in the regulation of fruit sugar accumulation in apple
Source: BMC Plant Biol. 2020 May 6;20:191. doi: 10.1186/s12870-020-02406-3 (PMC7203859; doi:10.1186/s12870-020-02406-3)
Supplement: Supplementary file 2 — Additional file 2: Table S2. Primer sequences of gene-tagged SSR markers for six MdSUT genes in apple. [file 12870_2020_2406_MOESM2_ESM.docx]

**Table S2.** Primer sequences of gene-tagged SSR markers for six *MdSUT* genes in apple

| ***SUT* gene** | **Primer (5'→ 3')** | |
| --- | --- | --- |
|  | Forward | Reverse |
| *MdSUT1.1* | GGAGTTCCTACTCATCTTTGATCC | TGAGAAAATGACAATCATATCCGTA |
| *MdSUT1.2* | CGGCGTGAGGATTCTTGTTG | ACACCGCCATCACCACCTC |
| *MdSUT2.1* | GGCCTATCTAGAATTTGGCACTCTC | TCAATTATGTAATGCTGCAGGCTG |
| *MdSUT2.2* | CTTTGGACCCCATTCCCTTC | GGGTGTCCCATAAAATGTGAAAG |
| *MdSUT4.1* | CACAATTCAAGGGCATTTCAGTC | TCTGCTTCTGGAGCTGGCAT |
| *MdSUT4.2* | AATTATTTGCCACAAATGCATGG | TTCCTGGTGCTAGTGCATCACAC |
