# Supplementary material for: The sucrose transporter MdSUT4.1 participates in the regulation of fruit sugar accumulation in apple
Source: BMC Plant Biol. 2020 May 6;20:191. doi: 10.1186/s12870-020-02406-3 (PMC7203859; doi:10.1186/s12870-020-02406-3)
Supplement: Supplementary file 3 — Additional file 3: Table S3. Sequences of primers used for vector construction. [file 12870_2020_2406_MOESM3_ESM.docx]

**Table S3.** Sequences of primers used for vector construction

| Gene | **Primer (5'→ 3')** |  | Note |
| --- | --- | --- | --- |
|  | Forward | Reverse |  |
| *eYFP* | GCAGGACTCTAGGGACTAGTCTTGTACAGCTCGTCCATGCCG | ACAATTACATTTACAATTACGGATCCATGGGCAAGGGCGAGGAG | Subcellular location |
| *MdSUT4.1* | CTCCTCGCCCTTGCCCATTGTGACAGCTCTGGGCTTTT | AATTACATTTACAATTACGATGCCAGCTCCAGAAGCAGACCGG | Subcellular location |
|  | TCGACACTAGTGGATCCAAAGAATTCATGCCAGCTCCAGAAGCAGACCGG | CATATCTCATTAAAGCAGGACTCTAGATCATGTGACAGCTCTGGGCTTTT | Transgenic analysis |
