# Supplementary figures and images for: The sucrose transporter MdSUT4.1 participates in the regulation of fruit sugar accumulation in apple
Source: BMC Plant Biol. 2020 May 6;20:191. doi: 10.1186/s12870-020-02406-3 (PMC7203859; doi:10.1186/s12870-020-02406-3)

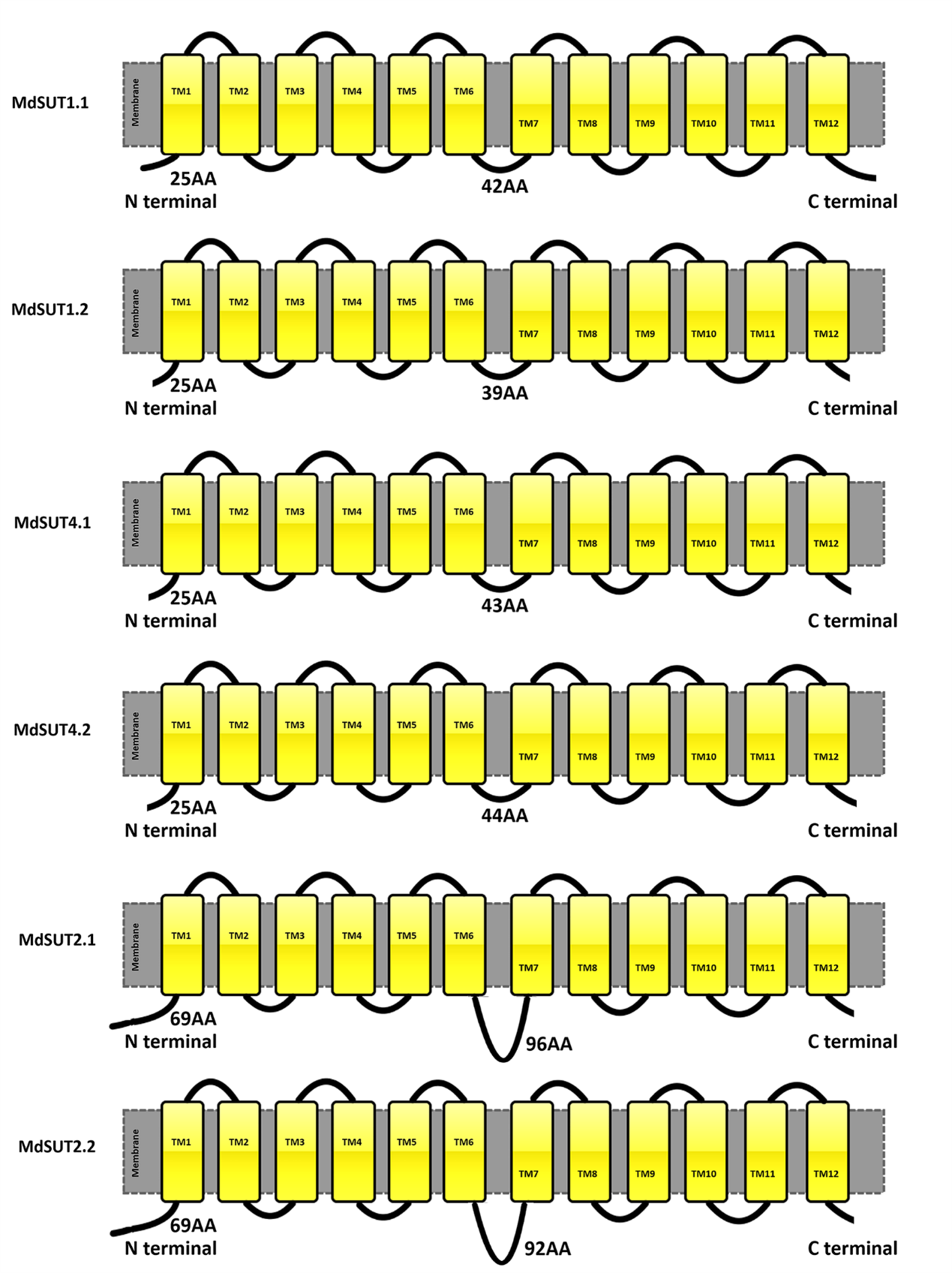


Fig. S1. A schematic of the predicted topology of MdSUTs in apple. AA, amino acid.

Supplement: Supplementary file 4 — Additional file 4: Figure S1. A schematic of the predicted topology of MdSUTs in apple. AA, amino acid. [file 12870_2020_2406_MOESM4_ESM.docx]
