# Supplementary material for: The sucrose transporter MdSUT4.1 participates in the regulation of fruit sugar accumulation in apple
Source: BMC Plant Biol. 2020 May 6;20:191. doi: 10.1186/s12870-020-02406-3 (PMC7203859; doi:10.1186/s12870-020-02406-3)
Supplement: Supplementary file 5 — Additional file 5:Figure S2. An example of genotyping of apple cultivars using SSR markers of the MdSUT genes. [file 12870_2020_2406_MOESM5_ESM.docx]

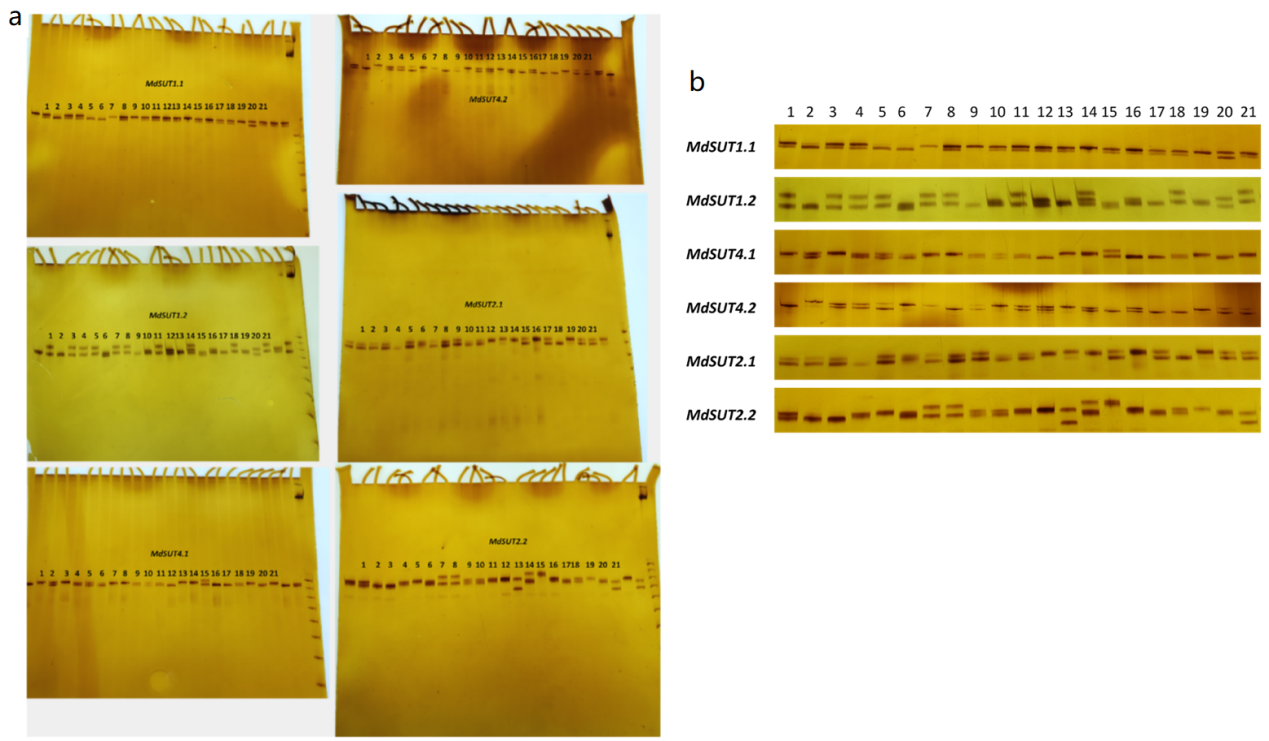


Fig. S2. An example of genotyping of apple cultivars using SSR markers of the *MdSUT* genes. a, the full image of polyacrylamide gel electrophoresis. b, the cropped version from the full image. 1, Cox Orangen Renette; 2, Millton; 3, Meltosh; 4, Generos; 5, Black Ben Davis; 6, Onieffnin; 7, Ingram; 8, King of pippin; 9, King David; 10, May; 11, Jinyu; 12, Italian Early; 13, Winter Banana; 14, Kuluona; 15, Tukumanpingguo; 16, Semei; 17, Early Harvest; 18, Guldborg; 19, Granny Smith; 20, Mishima Fuji; 21, Zaohong.
