# Supplementary material for: The sucrose transporter MdSUT4.1 participates in the regulation of fruit sugar accumulation in apple
Source: BMC Plant Biol. 2020 May 6;20:191. doi: 10.1186/s12870-020-02406-3 (PMC7203859; doi:10.1186/s12870-020-02406-3)
Supplement: Supplementary file 6 — Additional file 6: Figure S3. Alignment of vacuolar targeting di-leucine motif (LXXLL) in the N-terminus of SUT4 subfamily members in apple, peach, pear and Arabidopsis. [file 12870_2020_2406_MOESM6_ESM.docx]

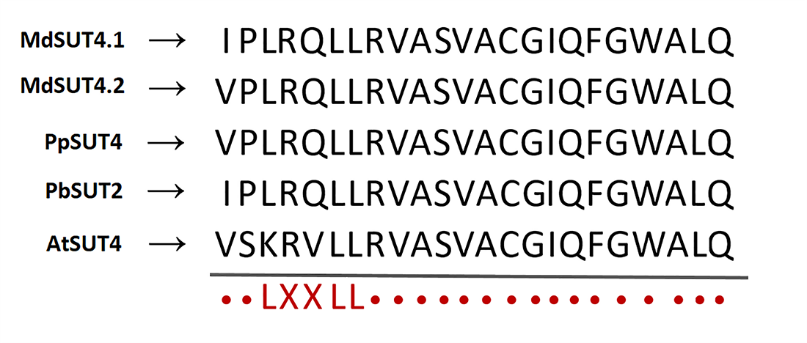


Fig. S3. Alignment of vacuolar targeting di-leucine motif (LXXLL) in the N-terminus of SUT4 subfamily members in apple, peach, pear and *Arabidopsis*. Red color letters represent consensus amino acids.
