# Supplementary material for: The sucrose transporter MdSUT4.1 participates in the regulation of fruit sugar accumulation in apple
Source: BMC Plant Biol. 2020 May 6;20:191. doi: 10.1186/s12870-020-02406-3 (PMC7203859; doi:10.1186/s12870-020-02406-3)
Supplement: Supplementary file 7 — Additional file 7: Figure S4. Expression of MdSOTs in apple calli overexpressing MdSUT4.1 (black column) and introducing entry vector pSAK277 (control, gray column). [file 12870_2020_2406_MOESM7_ESM.docx]

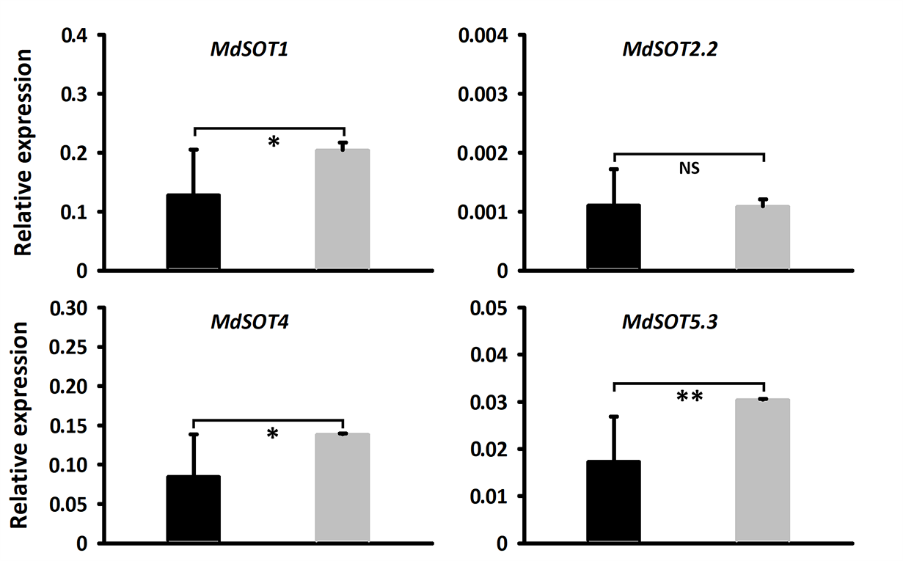


Fig. S4. Expression of *MdSOTs* in apple calli overexpressing *MdSUT4.1* (black column) and introducing entry vector pSAK277 (control, gray column). Statistical significance was analyzed by Student's t-test. **, *P* < 0.01; *, *P* < 0.05; NS, no significant difference (*P* > 0.05). Error bars represent standard error (SE) of three biological replicates.
